# “Anyone Know What Species This Is?” – Twitter Conversations as Embryonic Citizen Science Communities

**DOI:** 10.1371/journal.pone.0151387

**Published:** 2016-03-11

**Authors:** Stefan Daume, Victor Galaz

**Affiliations:** 1 Faculty of Forest Sciences and Forest Ecology, Georg-August-University Göttingen, Büsgenweg 5, 37077 Göttingen, Germany; 2 Stockholm Resilience Centre, Stockholm University, SE-10691 Stockholm, Sweden; 3 Department of Biodiversity Informatics, Swedish Museum of Natural History, Box 50007, 104 05 Stockholm, Sweden; University of Colorado, UNITED STATES

## Abstract

Social media like blogs, micro-blogs or social networks are increasingly being investigated and employed to detect and predict trends for not only social and physical phenomena, but also to capture environmental information. Here we argue that opportunistic biodiversity observations published through Twitter represent one promising and until now unexplored example of such data mining. As we elaborate, it can contribute to real-time information to traditional ecological monitoring programmes including those sourced via citizen science activities. Using Twitter data collected for a generic assessment of social media data in ecological monitoring we investigated a sample of what we denote biodiversity observations with species determination requests (N = 191). These entail images posted as messages on the micro-blog service Twitter. As we show, these frequently trigger conversations leading to taxonomic determinations of those observations. All analysed Tweets were posted with species determination requests, which generated replies for 64% of Tweets, 86% of those contained at least one suggested determination, of which 76% were assessed as correct. All posted observations included or linked to images with the overall image quality categorised as satisfactory or better for 81% of the sample and leading to taxonomic determinations at the species level in 71% of provided determinations. We claim that the original message authors and conversation participants can be viewed as implicit or embryonic citizen science communities which have to offer valuable contributions both as an opportunistic data source in ecological monitoring as well as potential active contributors to citizen science programmes.

## 1 Introduction

Social online media have emerged as important sources in the monitoring, prediction and modelling of trends and patterns in a broad range of domains. Commercial motivations drive many applications [1,2], as do political and sociological perspectives [3,4]. Applications include support in emergency situations [5,6] or better prediction of natural phenomena [7,8]. Some of the best researched and operational systems can be found in the domain of public health monitoring [9–11]. Increasingly, the potential of social media sources is also recognized in the environmental and ecological domain [12–15]. The volume, real-time nature and simple accessibility of this type of information source as well as advances in Big Data processing methodologies and tools, are major factors in support of this growing body of research on applications of social media mining.

An analysis of social media contributions with ecological significance could start by focusing on general mentions of environmental subjects interpreted as thematic trends [16]), but the pervasiveness of mobile devices with cameras combined with a broad set of social media channels provides great potential for real-time observations of ecologically relevant information [17] that may be contributed casually without knowledge of their ecological significance.

The value of such casual non-expert observations is underlined by an increasing number of so-called citizen science projects where members of the general public contribute to scientific research for example by providing or verifying biological observations [18]. This type of volunteer-driven monitoring contributes both to a wider coverage of monitoring efforts in general, and with the emergence of new technologies [19] may also have to offer more timely monitoring data compared to formal monitoring networks. Opportunistic biological observations in general therefore have the potential to contribute to early warnings of ecological changes [13], not least for potentially irreversible shifts in ecosystems [20]. At the same time, citizen science can serve as a tool to raise the public awareness for ecological changes and challenges [21], thus exhibiting characteristics not unlike social media which equally combine the profiles of data source and communication channel.

There is thus a large potential for ecological applications of this diverse set of social media information types. Compared to the prevailing themes in social media channels (such as music, entertainment, or news) specific ecological subjects may be marginal. However, the breadth of social media applications and the volume of major social media channels such as Facebook or Twitter hints at a significant amount of valuable information given the right tools [22]. Despite their acknowledged potential, very few tangible applications of these methodologies have been presented in the ecological domain to date.

### 1.1 Challenges: data quality and compatibility

One explanation for this scarcity of applications is that scholars and practitioners alike are rightfully sceptical about this type of data source. In contrast to professional ecological monitoring programmes social media data is unstructured, contributed outside a monitoring context, and exhibits known demographic and geographic biases [23]. This thus raises concerns about usability, representativeness, reliability and quality–the same concerns frequently voiced when the general value and impact of data generated by citizen science projects is discussed [24–26].

Examples from a broad range of domains can be cited to show that public participation in scientific research, can produce high quality data that serves as a valid basis for scientific results [25–28], although specific analytical tools [29] or adjustments through domain-specific contextual models [30] may be required. The growing interest in and importance of citizen science data has however led to a more thorough exploration of data quality, and rather than demanding standard formats and quality scales, approaches to formally capture quality and provenance as meta-data of a data set have been advocated [31,32]. Moreover, this should extend to “traditional” scientific data sources. A review of data managed as part of professional research workflows and large-scale data hubs [33] reveals for example that while of overall good quality it is not exclusively observational data sources originating from citizen science endeavours that struggle with incompleteness or exhibit errors and biases.

The concerns raised towards informal information sources such as social media are thus general, as these apply to varying degrees to all ecological data sources. While requiring specific tools and formal shared standards to ensure usability, it does however not preclude the usefulness of this data and its integration in the canon of ecological information sources. Given the volume of this data and the scope of current environmental challenges it seems thus both promising and critical to formally explore social media as data sources in ecological monitoring.

### 1.2 Social media, citizen science and ecological monitoring

Biodiversity observations are of particular interest from an ecological perspective. As a means to assess the usefulness and feasibility of social media as informal ecological monitoring sources, we collected a broad set of social media posts using the example of invasive alien species (IAS) in forest ecosystems. More precisely, we collected IAS mentions in messages posted on the micro-blogging service Twitter. These sorts of observations provide a good case to explore, as IAS are often not only highly visible for non-experts, but at the same time have well-known ecological impacts [34].

These Twitter messages include also cases of users seeking input from their social media networks in order to get clarifications or species determinations on original observations. Thus Twitter users posting requests for species identification and users in their networks answering these requests and providing species determinations show an interest and knowledge in environmental and biodiversity observations.

Biodiversity observations posted on Twitter and the ensuing conversations thus appear to align closely with ecological citizen science data, have to address the same quality concerns and exhibit activity patterns that fit common citizen science typologies [19,35]. Hence, ecological observations shared via social media may at least partially match the models and activities for public participation in scientific research, specifically the data-centric activities typical for “contributory”, “collaborative” or “co-created” projects which are frequently indicated as the most common models in citizen science typologies [35,36].

We therefore propose to explore and assess this instance of social media information in relation to citizen science data and activities, and moreover inquire whether these evolving and virtual small ad-hoc communities can be viewed as embryonic citizen science communities that could lead to active contributions to biodiversity monitoring. More precisely, we address two questions in this contribution:

1. What is the type and quality of the attainable social media data, specifically in relation to comparable citizen science projects?
2. What potential do these ad-hoc social media communities hold in engaging actively with citizen science projects?

## 2 Methods and Data

The data for this contribution was drawn from data originally collected for the *Ecoveillance* project [14], a research initiative that assesses the potential of online social media as informal sources in ecological monitoring in general and as providers for early warnings in particular. The project concentrates on Twitter as one social media source and focuses amongst other, on the example of invasive alien species. The *Ecoveillance* platform [37], developed as a web-based tool utilising the Twitter Search API, has been employed for the last three years to continuously obtain Tweets matching certain keywords that could indicate references to relevant ecological observations. The platform is currently not publicly accessible and used solely to support Twitter data collection for our research via concurrently active *monitors* that consist of predefined keywords.

Those keywords currently range from direct references to selected species (“oak processionary moth”, “emerald ash borer”), descriptive references (“hairy caterpillar”, “green bug”) or general observational statements (“I saw a moth”), resulting in a large pool of Tweets (currently approaching one million messages). While not specifically collected for the presented study we were able to identify a subset (N = 356) of message texts specifically indicating a request for a determination of a species and containing embedded media or linking to external media (e.g. Flickr or Instagram), thus Tweets containing phrases such as “anyone know what species”, “what kind of.” or “what type of …” (see S1 File for a complete list) and providing meta-data that could potentially support a determination of a species sighting. [38] is given as a representative reference to a sample Tweet triggering a Twitter conversation (replies by other Twitter users) and holding a broad range of the information we analysed (such as an image of an observed insect embedded in the Tweet, replies from other Twitter users with multiple suggested species determinations, including URL links to taxonomic verification resources and geo-location references). The full list of analysed Tweets is included as Supporting Information (S2 File).

It is important to note that both the subset of Tweets used for this study and the nearly one million Tweets matched by the Ecoveillance platform will represent only a small proportion of all Twitter messages that could be classed as relevant biodiversity observations. Firstly, we concentrated only on English language keywords as search terms, thus limiting the geographic and demographic coverage of the obtained messages. Secondly, both the public Twitter Search API (Application Programming Interface) utilised by the Ecoveillance platform and the alternative Twitter Streaming API provide access to a small share of all potential Tweets; informal estimates for the coverage of these APIs vary significantly with some sources stating that for example the Twitter Streaming API provides a 1% sample of all Tweets in real-time whereas the coverage via the Twitter Search API depends on a combination of a search term’s frequency and popularity since this API is geared towards popularity rather than completeness. Operational systems should pursue alternative, and certainly computationally more resource-intensive, approaches to obtain matching data and estimates of the abundance of this information, should cover other languages and apply search terms that specifically target requests for a species determination.

We further filtered our dataset, by removing duplicates and excluding Tweets that themselves were no longer accessible (or essential resources they were linking to, i.e. media links, user profiles), which left us with 215 unique Tweets for analysis. All classification tasks presented throughout this study were done manually, supported by a classification module integrated in the *Ecoveillance* platform.

In an initial classification we concentrated on deciding whether these Tweets with the above matching phrases were indeed “on-topic”, thus whether they represented examples for biological observations with a request to a Twitter user’s network for a taxonomic determination of the observed species. The results are summarised in Table 1, which also provides information on the interpretation basis of the “on/off-topic” categorisation. While it is primarily the textual content of a Tweet that allows a decision on topical relevance, this is not exclusively the case. The decision basis for topical relevance is of importance when considering a future automatic approach to obtaining, classifying and analysing such Tweets and conversations–if textual content (Tweet messages, user profiles) suffices for this classification, automatic processing can be deemed more feasible.

**Table 1:** Number of "On-topic", "Off-topic" and “Undecidable” Tweets and the required information items that contributed to the determination of the topical relevance. The interpretation basis percentages in each row add up to more than 100% as multiple information items may have contributed to the decision on topical relevance. Linked URLs, user profiles and ensuing conversations were also considered as a potential interpretation basis, but were not required for this dataset.

N of Tweets		Interpretation basis (%)		
		Tweet text	Embedded media	External media
**On-topic**	191	99.5	11.5	8.4
**Off-topic**	22	95.5	9.1	-
**Undecidable**	2	-	-	-

The 191 “on-topic” Tweets with embedded or linked media and (where applicable) conversations were subjected to further analysis. Specifically, we

- assessed the quality of the embedded or linked media with regard to a likely determination of the observed and imaged species,
- extracted textual references to geo-locations in the Tweets, geo-coordinates attached to the Tweets and location information provided in Twitter user profiles,
- noted whether the posted Tweet triggered a conversation, how long it was and where it took place (Twitter or external media such as Instagram or Facebook),
- whether the conversation included one or more answers to the requested species determination, what level of taxonomic detail it covered, who provided it and if it was (as far as determinable) correct or not,
- and finally what type of environmental background the requesting and answering Tweet authors had.

Furthermore, we utilised the rich metadata for each Tweet—accessible through the Twitter API—to obtain additional information of relevance such as geo-location information associated with Tweets and user profiles, size of a user’s network (“followers” and “friends”) or the number of Twitter user’s mentioned in a Tweet. The applied categories and utilised metadata will be explained in more detail in the relevant results sections.

## 3 Results

### 3.1 Conversations

In an initial assessment each of the 191 “on-topic” Tweets was reviewed for the occurrence of conversations on either Twitter or social media sources linked from the Tweet (specifically Instagram, Flickr, Facebook). Table 2 summarises the results of this analysis.

**Table 2:** Conversations identified for the analysed data set, showing total number and shares by conversation medium and reply type (i.e. with or without species determinations). A ‘determination reply’ is a conversation that contains at least one reply that suggests a taxonomic determination of the posted biodiversity observation. Shares add up to more than 100% as some Tweets received parallel replies on multiple media.

Conversation	None	Twitter	Instagram	Facebook	Other	*∑*
**No reply**	**69** (36.1%)					*69*
**General reply**		**12** (6.3%)	**5** (2.6%)	**1** (0.5%)	**1** (0.5%)	*19*
**Determination reply**		**77** (40.3%)	**35** (18.3%)	**2** (1.0%)	**1** (0.5%)	*115*
***∑***	*69 (36*.*1%)*	*89 (46*.*6%)*	*40 (20*.*9%)*	*3 (1*.*5%)*	*2 (1*.*0%)*	

Overall, 64% of all Tweets analysed are answered, and 86% of those conversations do contain at least one reply providing a species determination in response to the original Tweet author’s request. Twitter and Instagram, an image sharing tool with options to reply to posted images, are the primary conversation media, with no significant difference in the share of replies with determinations. In 12 instances parallel conversations on Twitter and Instagram could be observed, but the majority of conversations happen exclusively on one medium, mostly on Twitter.

### 3.2 Observational characteristics

Each Tweet analysed in this contribution represents a unique biological observation. In the following sections we provide an overview of the media type and quality, temporal patterns, geo-information and source meta-data associated with these observations. This will help assess the type and quality of the attainable data.

#### 3.2.1 Media type and quality

**Fig 1:**
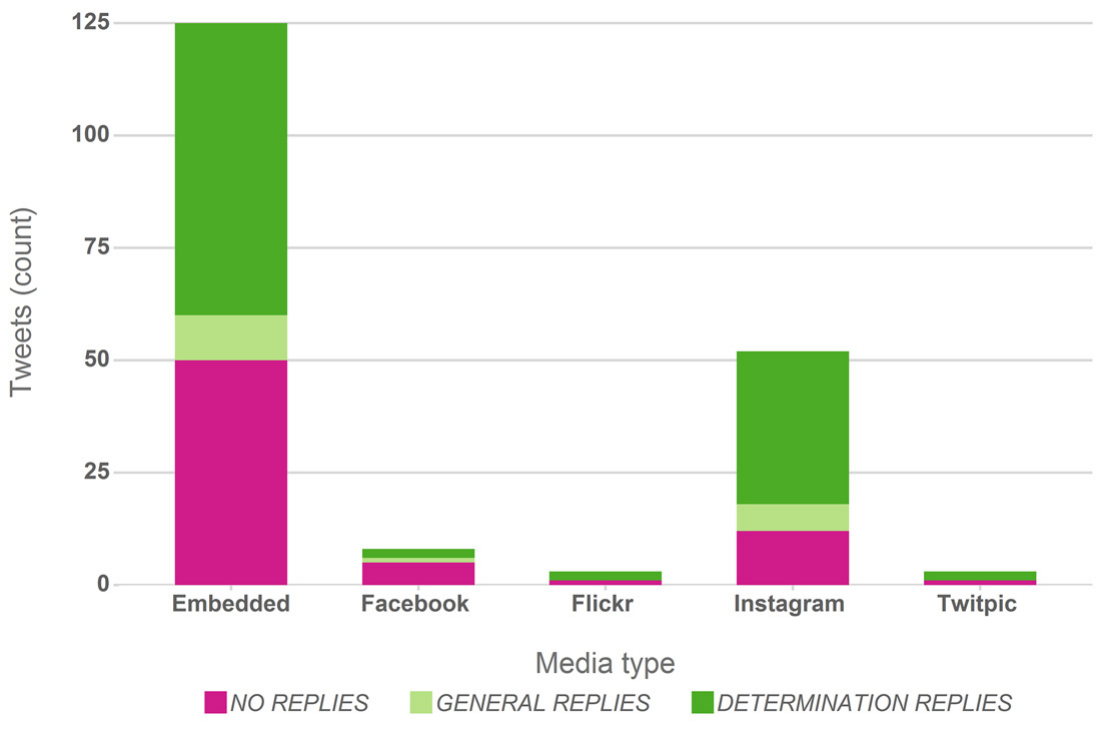
Type of embedded or external media associated with the analysed Tweets and type of reply. ‘Embedded’ media are images that are shown embedded within the Tweet text. External media appeared as URL links to Facebook, Flickr or Instagram in the Tweet text. The share of unanswered Tweets (orange), Tweets receiving replies with (green) and without (blue) suggested taxa is highlighted for each media type.

All analysed Tweets contained images as the sole shared media type rather than videos or sound files, which are also frequently shared on Twitter or other social media sites. Fig 1 summarises the found image types, highlighting that the majority (65%) are embedded in the Tweet, thus visible directly to a user viewing the post. This may in fact apply to other media as well such as Flickr and Twitpic, which have only negligible shares though. Images shared through Instagram and linked from the Tweet account for approximately 27% of all posted images.

Aside from relevant expertise in a Tweet author’s network a key factor in receiving determinations will be the quality of the posted images. Images of higher quality will be more likely to support a conclusive identification of an imaged species. With a view on the likely determination of the imaged species the quality of all images posted with the 191 “on-topic” Tweets was thus assessed manually and assigned to five quality classes ranging from *“very poor”* to *“very good”* with the former assuming that a determination may be near impossible while for the latter a species determination was assumed near certain if an expert would be given access to the picture.

Factors contributing to a higher quality and in turn likelihood for species determination are the general quality features of an image (resolution, lighting, sharpness, contrast, colour space), the relative size of the photographed species, distinctiveness of the species itself as well as the availability of direct or indirect scales, helpful peripheral information or textual content that provides context to the image and the captured species, such as for example mentions of colours or geo-locations. Hence, the lower resolution of an image may for example be compensated by the availability of geo-location information or the visibility of a species’ distinguishing features. However, since quality was assessed as a combination of properties that could support a species determination, these quality classes may overlap to some extent, and the quality assignments should thus be seen in conjunction with the assessment of determination correctness (section 3.3).

**Fig 2:**
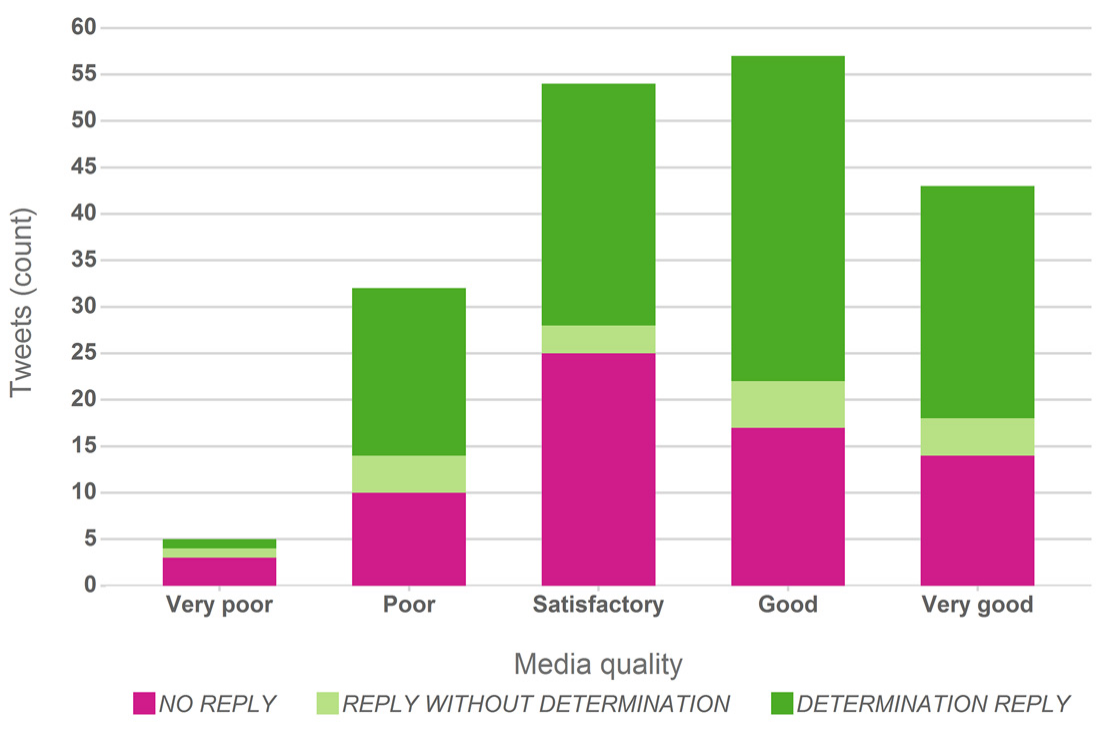
Quality of the posted media with regard to a likely determination of the captured species distinguished by type of reply. The share of unanswered Tweets (orange), Tweets receiving replies with (green) and without (blue) suggested taxa is highlighted for each quality class.

Fig 2 summarises the results of this quality assessment, again distinguishing between images that received no reply, general replies and determination replies. Overall, the majority (81%) of shared images were of satisfactory or better quality thus lending itself to verify or identify an observed species.

#### 3.2.2 Temporal characteristics

**Fig 3:**
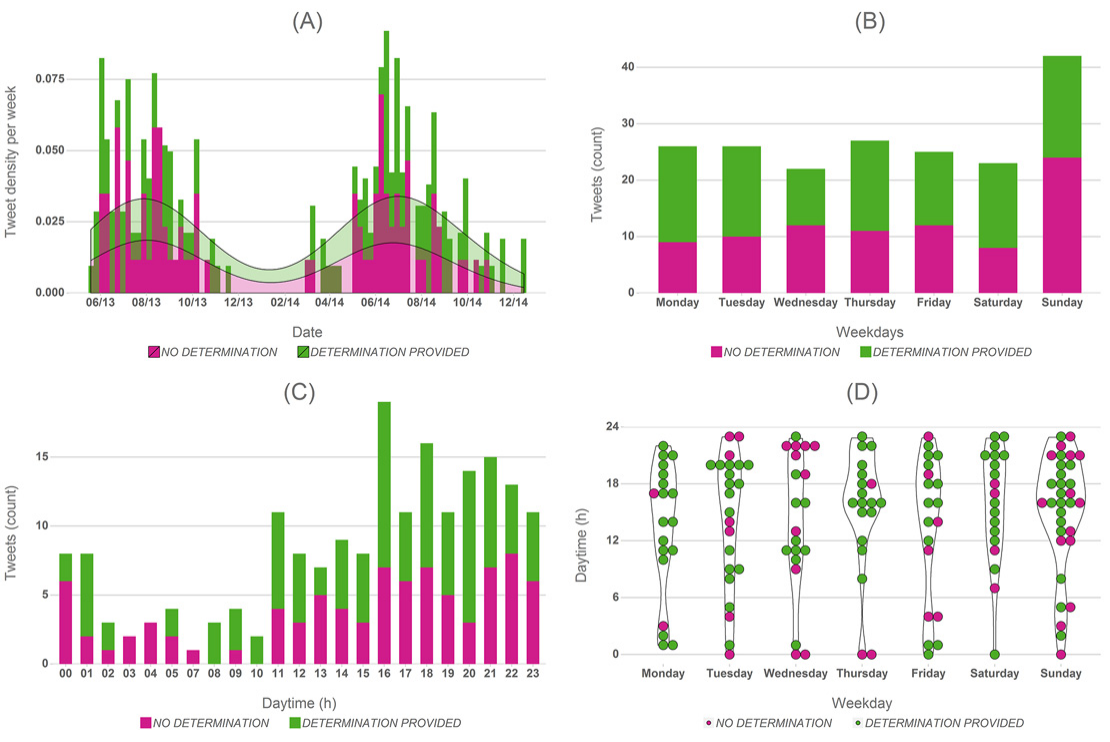
Temporal characteristics of the analysed Tweets. Tweet frequencies (A) by week in the data collection time period, (B) by weekday, (C) by hour of day and (D) as a combined distribution over weekday and daytime.

The analysed “on-topic” Tweets were posted during the period from May 2013 to December 2014. Fig 3(A) shows the weekly frequencies of these Tweets in the data collection timeframe. For a start, the date distribution actually underlines the bias in our data. Since we used Tweets for this analysis that were originally collected with a focus on invasive alien species in forest ecosystems, the distribution is seemingly a reflection of the lifecycle of the originally targeted species, rather than necessarily the observational activity of the authors posting the photos and requesting determinations. While our original sampling focus and sample size may not allow a generalization, it is however fair to assume that the type of casual observation we analysed here are more likely made during core lifecycle phases of the observed organisms, not least because daylight and weather conditions will probably coincide with general and recreational outdoor activities of the potential observers.

Fig 3B–3D illustrates that the posted observations exhibit some interesting additional temporal features. Based on the content and wording of the analysed Tweets, we can assume that these observations are casual rather than deliberate monitoring events. The weekday distribution of the posted Tweets confirms this (Fig 3(B)), with a clear spike on Sunday (22% of all Tweets), thus a day where people can generally be expected to be off work and engage in recreational activities. While a similar pattern may be expected for Saturday, it is very pronounced for Sunday.

Looking at the daytime distribution of the Tweets (Fig 3(C)), it is not surprising that there is little activity at night-time and early morning. A slight peak can be observed around lunchtime, again suggesting the opportunistic and casual nature of observations probably made during lunch breaks; this is confirmed by the combined weekday/daytime distribution in Fig 3(D), which also underlines that the daytime distributions are similar across all weekdays. However, it is notable that many observations are posted late in the day and evening (Fig 3C and 3D), even more so given that the review of the pictures suggested that they were almost exclusively taken in daylight. There is thus a notable reporting latency and the Sunday reporting peak could also be attributed to observations made on Saturdays and reported with a latency of a whole day rather than just few hours. However, the pronounced reporting peak at the weekend seems to support a reporting latency of up to 24 hours rather than several days or weeks, and significantly larger latencies (weeks or months) can be excluded given the majority of observed species (see also section 3.3) and the good fit of the observation frequencies with the lifecycles of those species (Fig 3(A)).

Hence, while pictures were apparently taken casually in daylight, the latency in posting the request for determination suggests a more than casual interest in the subject. We will revisit this observation when discussing the potential of the Tweet authors’ contributions in the context of citizen science in general.

#### 3.2.3 Geo-location information

With few exceptions, biodiversity observations will be of value only in connection with geo-location information, that ties those observations to a specific ecological monitoring context (e.g. invasive species detection) or makes them useable as input to ecological models (e.g. species spread and distribution). Twitter applications typically offer a mechanism to attach geotags (detailed geo-coordinates) to a posted Tweet, which are then available as part of the Tweet metadata via the Twitter API. These geotags will be of particular quality and accuracy when Tweets are sent from GPS-enabled mobile devices.

However, of the 191 analysed Tweets only two were available with geo-coordinates. This is in line with results in other studies. [39] found that the share of Tweets with geo-coordinates is typically in a range of 0.5% to 3%, but depending on the studied subject and messages’ geographic origin the proportion of precisely geo-tagged Tweets can be significantly higher; [8] cite several studies where geo-tagged posts accounted for 5% to 16% of collected Tweets.

**Fig 4:**
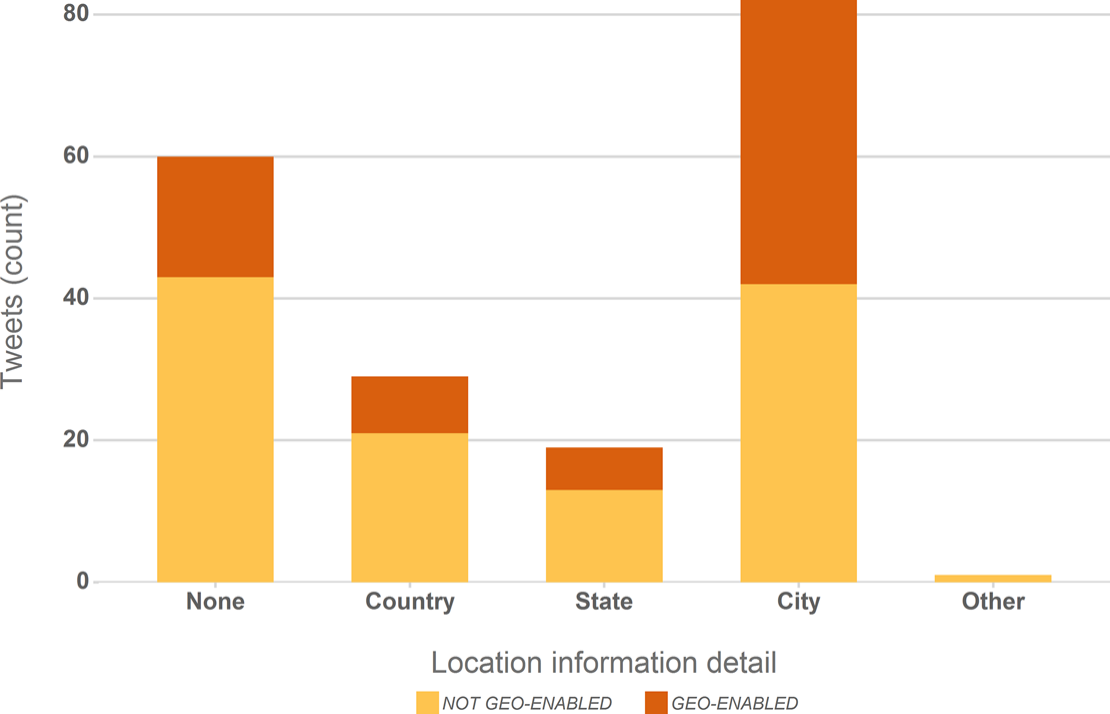
Granularity of location information in user profiles for the analysed Tweets and share of geo-enabled user profiles in each location category.

Geo-information, albeit less accurate and reliable, is however available in other forms as well: as volunteered location information in a Twitter user’s profile or as textual references. User profile location information is obtainable through the Twitter API. Fig 4 summarises the granularity of the available geo-location information in user profiles of the authors of the analysed Tweets. Nearly one third (31%) of users do not provide usable location information in their profiles, but 43% of user profiles hold locations at the granularity level of “City”, which may still cover a large region (New York, London), but narrows the geo-placement of the observed species. This again matches results in other studies: both [39] and [8] report that descriptive toponyms associated with analysed Tweets vary within an equally broad range (21–70%). However, there is no guarantee that Tweet authors took the posted images at their profile location and profiles may get out of sync with a Twitter user’s actual location.

Textual location references were found in 20% of analysed Tweets. While more reliable they exhibit a similar granularity (ranging from e.g. “USA” to “Rainham Marshes” or “Ashford train station”) and require a similar validation and mapping step if extracted automatically from the message text.

Fig 4 highlights another characteristic with regard to Twitter geo-information: the proportion of Tweet authors with “geo-enabled” profiles, a Twitter platform setting that triggers the automatic geotagging of Tweets. Interestingly, 37% of all analysed Tweet authors and 49% of those providing a “city” in their user information had geo-enabled profiles, and yet only two Tweets in our dataset carried geo-coordinates. The most likely explanation is that while user’s geo-enabled their Twitter profiles (which is not the default setting), they did not geo-enable their devices or blocked geo-tagging on those devices for certain applications. We can only speculate if this is a deliberate choice for the specific Tweets we analysed or if this setting merely was forgotten resulting in the lack of geo-coordinates. We nevertheless can observe that the authors in our dataset must have made a deliberate choice to geo-enable their profiles. Thus, while geo-information is lacking or does not propagate through, if settings on the used devices were in sync with this choice we could expect a large amount of observations with high-resolution geo-information.

#### 3.2.4 Tweet source devices and applications

**Fig 5:**
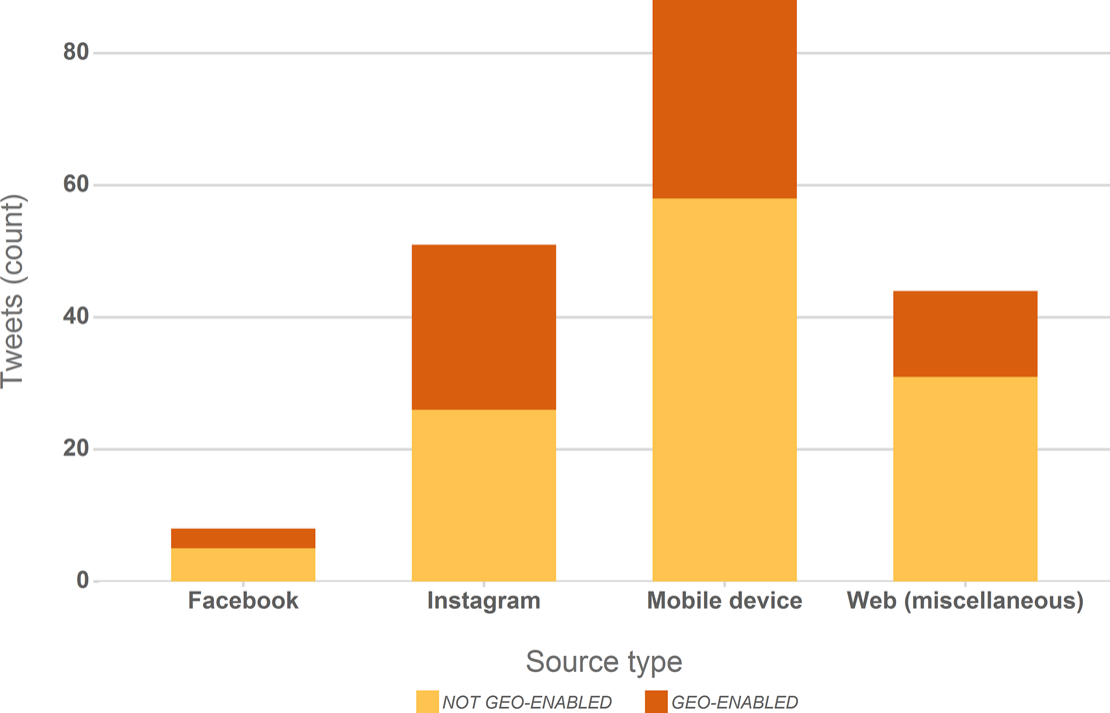
Source devices and applications from which the analysed “on-topic” Tweets originated with an indication of the share of geo-enabled profiles associated with Tweets in each category.

A look at the prevalent source devices and applications of the analysed Tweets (Fig 5) underlines the point made in the previous section; information on the source device or application is part of the metadata obtainable for a Tweet via the Twitter API. We summarised instances clearly identifiable as originating from *Mobile devices* (e.g. Blackberry, iPhone, Android) or *miscellaneous web* applications (e.g. Twitter website, TweetDeck) in two classes, the remainder originated from Twitter integrations of Instagram or Facebook.

Mobile devices were clearly identifiable as sources of the postings for 46% of the posted Tweets. The actual number is however almost certainly significantly higher, since Facebook or even miscellaneous web applications could have been used from mobile devices and Instagram (27%) is geared towards mobile usage. We can thus assume that the potential rate of geo-tagged Tweets could be much higher if a user would choose to share geo-coordinates from a mobile device together with the published Tweet.

### 3.3 Species determinations

**Fig 6:**
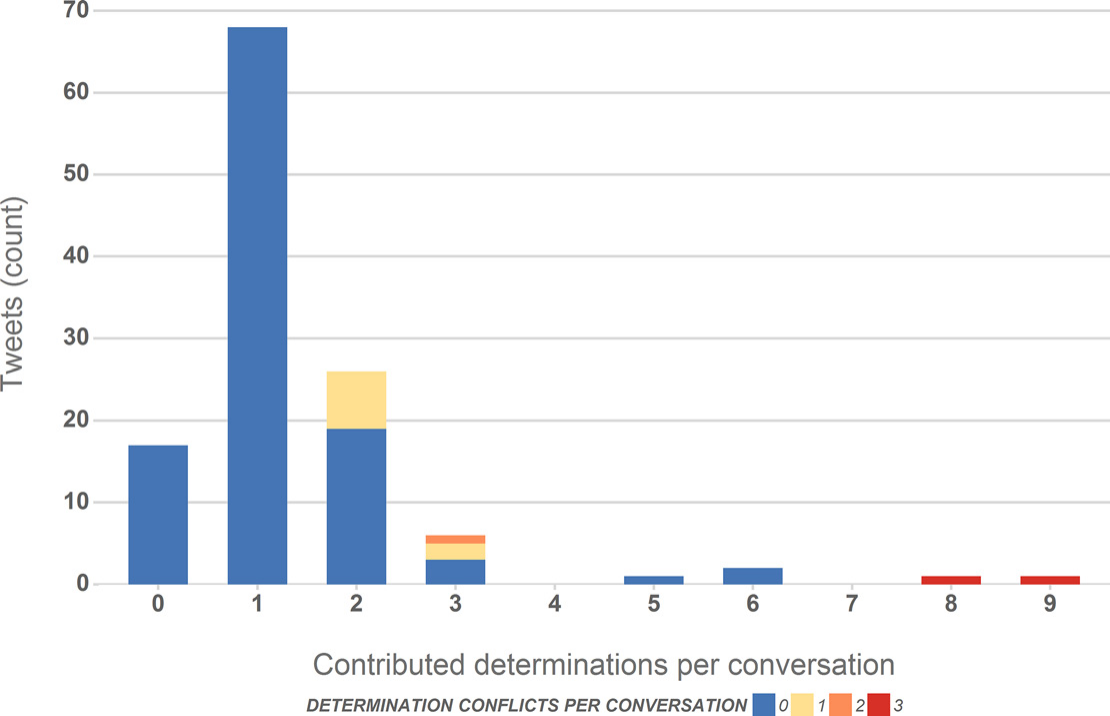
Number of suggested taxa (determinations) per Tweet for Tweets receiving replies and number of conflicting determinations. The share of Tweets with conflicts and the number of conflicts is indicated with a red colour scale.

In Table 2 we distinguished between conversations with general replies and those containing at least one suggested determination for the species a Tweet author photographed–in 86% of all conversations at least one of the participants provided a determination. Fig 6 explores the number and nature of these determinations in greater detail. The majority of conversations (56%) contain only one suggested determination. Of the 37 conversations with more than one determination 32% contain alternative determinations. We consider those “conflicts” as resolved, if the contributing conversations authors settle on one determination or at least one of the determinations is correct, which applied in all but 4 instances.

On closer inspection, these “determination conflicts” or longer determination conversations represent valuable information by itself since they capture a vetting process that can be interpreted as explicit meta-data on the reliability of the information. Sometimes these conversations take the form of singular determination statements, sometimes additional information is requested and provided, leading to improved determinations. Moreover, these type of conversations offer contextual information that will not be available in standard biodiversity observation databases, for example when contributors express surprise about a sighting at a particular location or outside an expected time window, mention the rarity or commonness of a species, or comment on the reliability of a determination in the context of geo-information, lifecycles or other environmental contexts. All these variations were represented in our dataset, but given the size of the available Tweet sample are illustrative and do not yet permit provision of a detailed profile of this interesting contextual meta-data.

**Fig 7:**
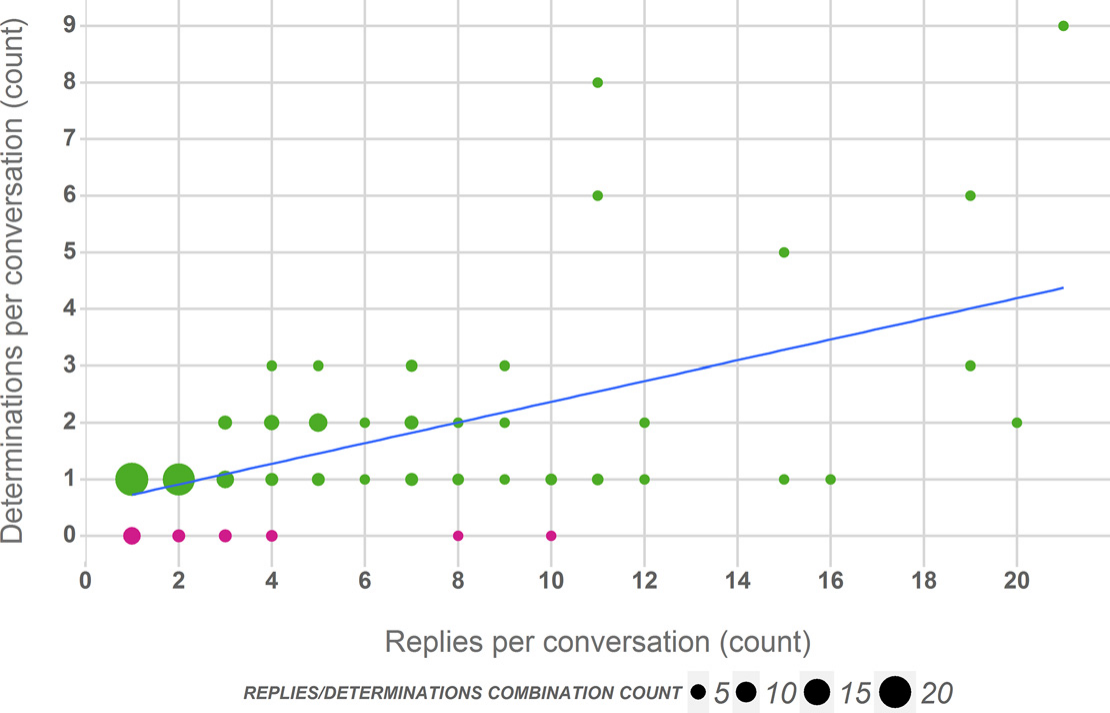
Relation of number of replies containing a species determination to the total number of replies per analysed conversation. For readability orange circles were used to highlight conversations without determination replies as opposed to conversations with determination replies (green). The size of the circles indicates the frequency with which a specific combination of reply and determination counts was found.

Fig 7 illustrates the relation between the number of replies and determinations per conversation, which expectedly suggests that longer conversations contain more determination replies. This trend is however not very pronounced. As Fig 6 illustrated as well, the majority of conversations are short and only have one or two determination replies. This observation could be explained by either assuming that the authors requesting a determination only have access to a small pool of experts in their network or that a provided determination reduces the motivation for others to contribute an additional answer.

**Fig 8:**
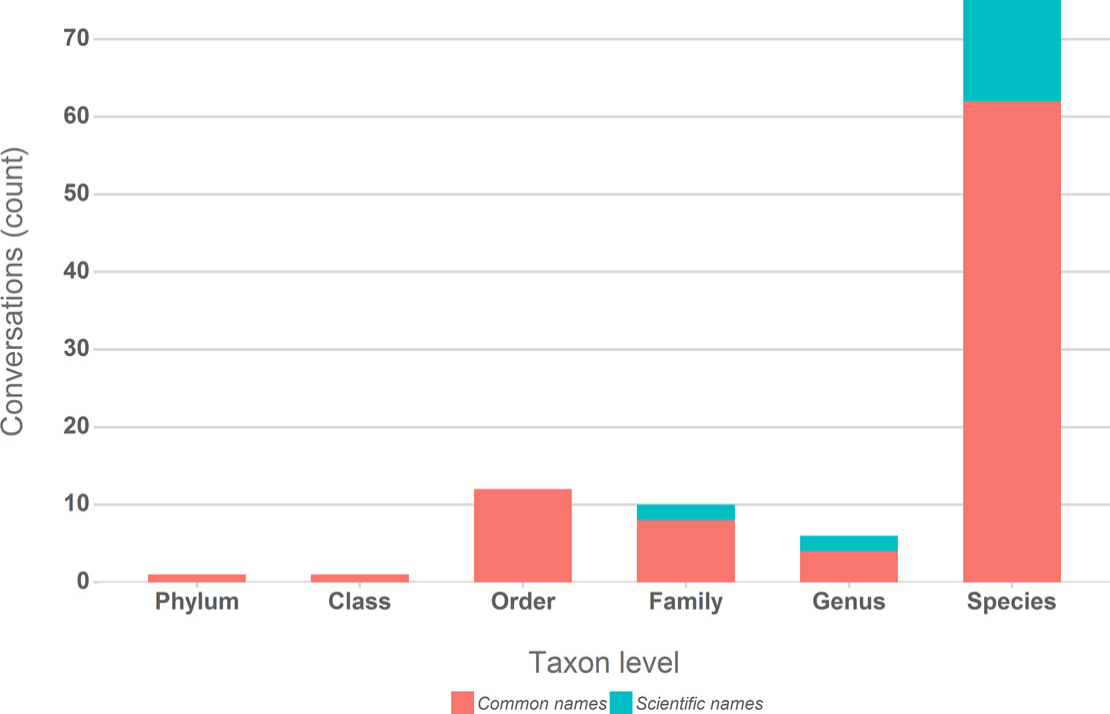
Highest taxonomic detail and choice of terminology (scientific or common names) for provided determinations per conversation.

**Fig 9:**
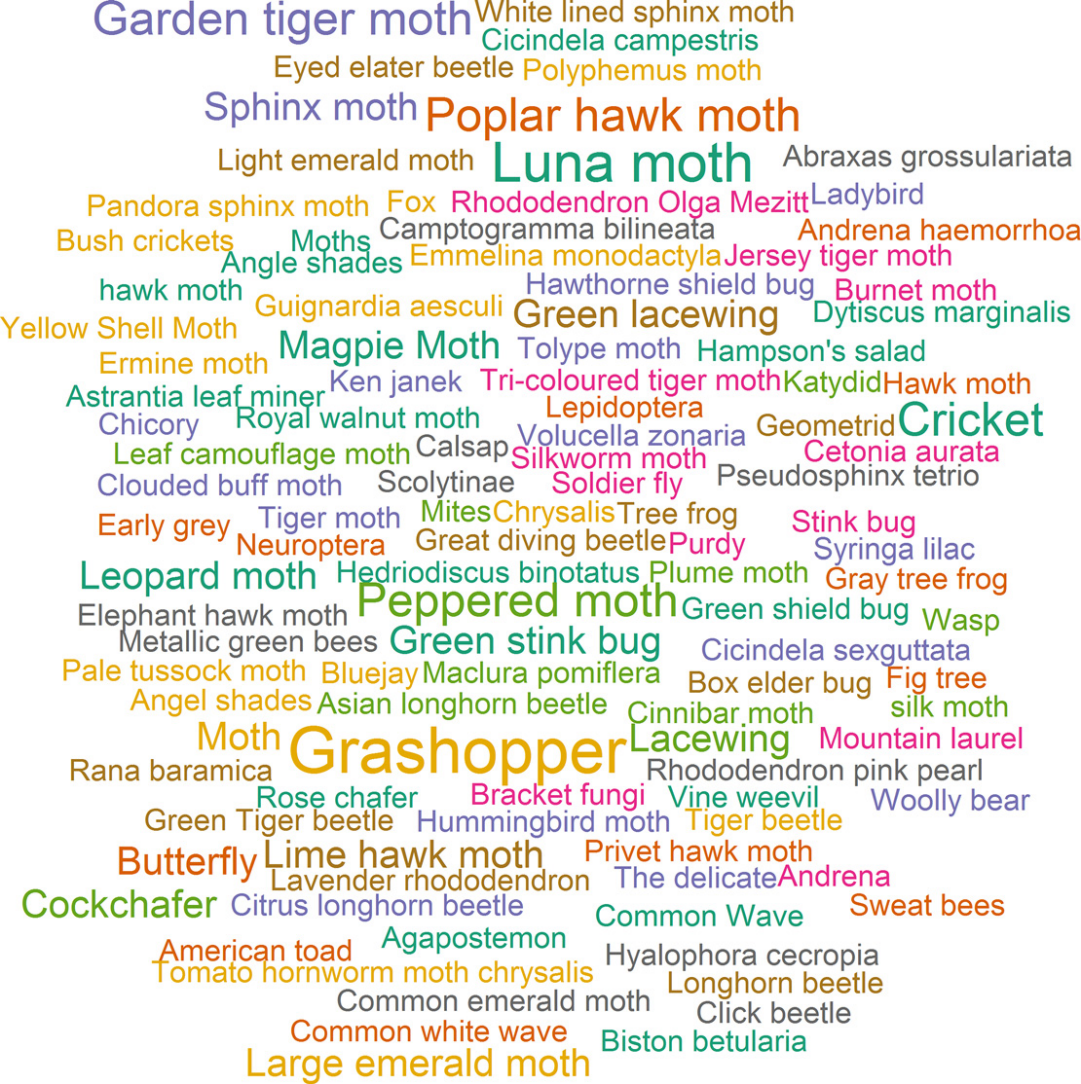
Word cloud showing all provided determinations in the analysed conversations. The word size indicates the frequency of suggested common or scientific species names, ranging from a maximum of five mentions (‘Grashopper’) to single mentions (e.g. ‘Cicindela sexguttata’).

In a further analysis of the provided determinations we noted the level of taxonomic detail, the used terminology and the actual provided determinations. Fig 8 summarises the highest taxonomic level provided for each conversation with determinations and whether common or scientific names were used. In 71% of all conversations the request is answered with a determination at the species level. Only in 12% of cases however the determination providers contribute determinations using scientific names. In 16% of conversations the determination providers back up their claim with a link to taxonomic references such as for example *ukmoths*.*org* or *Wikipedia*. Fig 9 is included for illustration, quoting all determinations provided in the analysed conversations.

In a final evaluation of the provided determinations we followed up the claims and tried to assess if a determination conversation resulted in a correct determination (Table 3). Some of these assessments had to be marked as uncertain, due to the quality of the posted images, limited visibility or lack of distinctive features of the assessed organisms as well as the authors’ taxonomic expertise.

**Table 3:** Assessment of correctness of contributed taxonomic determinations in the analysed conversations. **The numbers in parentheses indicate assessments where correctness could not be decided with absolute certainty.** For the two instances assessed as ‘partially correct’ only the *Genus* could be confirmed as correctly determined.

Determination assessment	Correct	Partially correct	Incorrect	Undecidable
**Conversation count**	80 (29)	2 (1)	9 (7)	14
**Conversation %**	76.2% (36.3%)	1.9% (50.0%)	8.6% (77.8%)	13.3%

Overall the quality and reliability of the provided determinations can be assessed as high. With caveat of the noted uncertainty margins, only 9% of the determination conversations produced incorrect results while 78% were correct or partially correct.

### 3.4 Contributor classification

In order to reflect on contributors to the analysed Tweet observations and determination conversations in the context of citizen science, we carried out a categorisation of the original Tweet authors and users providing determinations. Our classification scheme was motivated by the question whether the two groups of observation and determination contributors are dominated by contributors with a documented environmental interest, education or profession.

We included all users (N = 191) contributing observations (including unanswered ones), and all users (N = 114) providing determinations in Twitter conversations. Users contributing determinations in Instagram conversations were not included because the user information accessible on these sites did not provide a sufficient basis to assess the background or interest of the users. For Twitter users their environmental interest or formal domain education was assessed manually based on their Twitter user profiles, external sites linked from those profiles and the content of their Tweets in general; despite this typically large amount of direct and contextual user information a margin of error in these classifications naturally remains, for example if users maintain strictly separate private and professional accounts. Table 4 provides a complete list and explanation of the applied author classes.

**Table 4:** Classification scheme applied to all Twitter authors requesting and providing species determinations in the analysed Tweet set.

Class	Classification criteria
**Domain professionals**	Individuals with a formal education and/or profession within the environmental or biological domain including for example researchers, foresters, farmers or professional gardeners, etc.
**Amateur biologists**	Individuals with a specialised biological subject interest (entomology, ornithology) pursued as a recreational activity but following professional standards and methods. This includes individuals with a documented participation in citizen science projects.
**General nature enthusiasts**	Individuals with a strong documented personal but not professional interest in nature and outdoor activities (e.g. gardening, photography), including environmental activists.
**Environmental organisations**	Organisations with a documented association to environmental or biological subjects, including research organisations, conservation groups or gardening associations, entomological or ornithological societies, etc.
**Social media aggregators**	Special Twitter channel dedicated to “retweeting” Tweets by other users reporting biological observations.
**Miscellaneous organisations**	Miscellaneous public or private organisations, including companies, with no discernible environmental background or domain function.
**“Incidental” biologists**	Contributors of the analysed Tweets or conversations with no discernible domain background or documented environmental activities.

**Fig 10:**
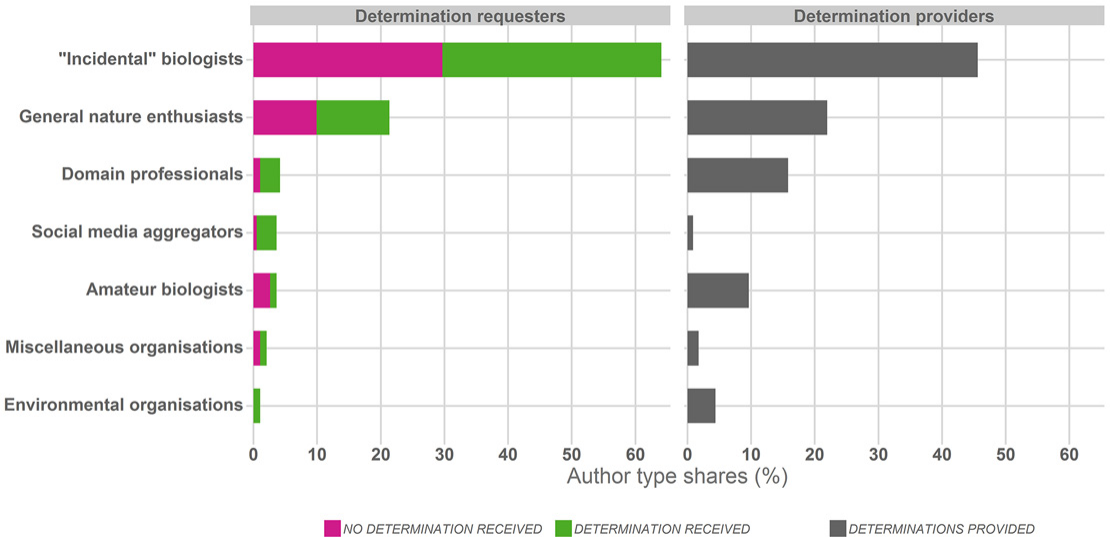
Type of users requesting determinations and providing determinations, with categories indicating contributors with a documented environmental interest, education or profession if any. The classification of users is based on available Twitter profiles, linked personal pages and the content of Tweets authored by them; *“Incidental” biologists* denote users with no discernible biological/environmental background or activities.

Fig 10 compares the shares of the different contributor types in the two groups of Twitter authors posting observations and requesting species determinations, and those providing determinations. Both groups are dominated by individuals, organisational contributors account for a marginal share only. Furthermore, in both groups contributors with no discernible environmental background (denoted *“Incidental biologists”*) represent the largest share, 64% of determination requesters and 46% of determination providers. The second largest contributor type–with 21% and 22% respectively–are those termed *“General nature enthusiast”*. Very few individuals (8%) with a professional or quasi-professional domain background (*“Domain professional”*, *“Amateur biologist”*) request determinations but this group accounts for 25% of all provided determinations.

**Fig 11:**
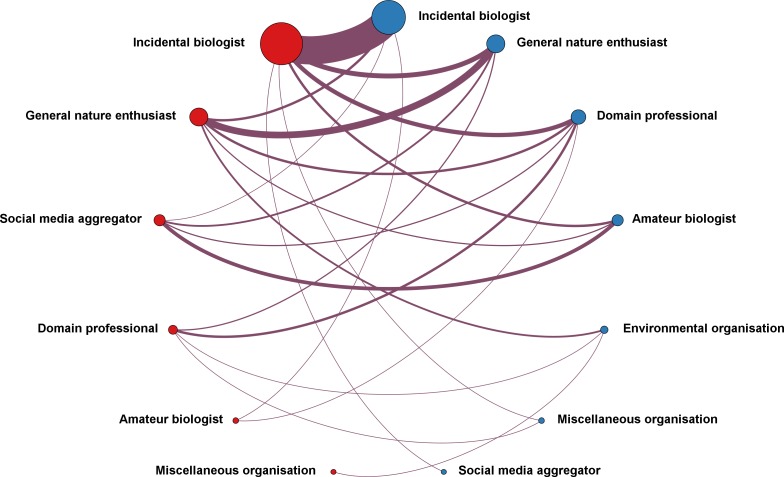
Connections between different types of determination requesters and providers. Red circles represent user types requesting and receiving determinations, blue circles user types providing determinations. The size of the circles indicates the frequency of an author type, the size of the edges the frequency with which a particular pairing can be found. The graph was generated with the Gephi (https://gephi.org) network visualization tool using a Circular network layout.

Fig 11 adds an additional dimension to the contribution of different author types requesting and providing determinations. The request for and provision of determinations is represented as a network that captures the frequency of author types and the frequency of certain combinations of author types.

As already noted in Fig 10 the largest group are Twitter users with no discernible environmental background (*“Incidental biologists”*). They also represent the most abundant connection, thus requests by users with no documented environmental background are answered by the same type of users. This may not be surprising considering the dominance of this user type in our dataset, but notable when considering the large proportion of correct determinations.

The second most abundant connection is between the contributor classes termed *“General nature enthusiast”*. We can observe that determination requests by this contributor type are primarily answered by the same type of contributors. At the same time *“General nature enthusiasts”* are the second most frequent determination providers to users with no environmental background (*“Incidental biologists”*).

Interestingly, we can also observe that if users with a formal or professional background ask for determinations (*“Domain professionals”*) they are predominantly answered by users with a similar background (*“Domain professional”*, *“Environmental organisation”)*. It can be argued that this should not be surprising since connections in Twitter networks will be driven by shared interests, thus researchers can be expected to have a large share of other researchers in their network (see for example [40]). Twitter networks will not be completely homogenous though and one can ask if non-experts generally tend to assume that their contributions will have no added value for experts, and that non-experts are thus less likely to provide species suggestions to experts. Considering however the abundance of non-experts providing determinations and the overwhelming correctness of those, we can speculate if an even larger contributory potential from non-experts remains unutilised. A definitive and quantitative assessment of the contributory potential of non-experts as well as the self-assessment of their domain expertise will however require a direct engagement with Tweet authors through survey-based future studies.

### 3.5 Results summary

We analysed 191 Tweets with biodiversity observation posted with a species determination request, 64% received replies, 86% of those contained at least one suggested determination, of which 76% were assessed as correct. All posted observations included or linked to images with the overall image quality categorised as satisfactory or better for 81% of the sample and leading to taxonomic determinations at the species level in 71% of provided determinations.

While acknowledging that we used a dataset originally collected for another purpose and thus working with a comparatively small sample, the above summary of some of the main results suggests that we are dealing with a valuable resource both with regard to the published biodiversity observations as well as the contributions of the participating community. Importantly, this data can be considered as lost since it is published outside an ecological monitoring context and channel, thus not collected, assessed and utilised, which highlights the potential contribution of this data source in ecological monitoring efforts.

## 4 Discussion

One of the key features of observational data obtained via social media channels such as Twitter, Facebook or Instagram is its real-time nature. In light of a recent critique of shortcomings of traditional ecological monitoring programmes [41] the value of real-time monitoring data in particular can be stressed, and an exploration of social online media as additional data sources in ecological monitoring seems merited as it may help to address not only issues such as timeliness, but also contribute to question-driven monitoring [14,41].

This type of data can be considered even more valuable if it extends beyond plain and undetermined observations and is instead vetted and reviewed, thus possibly approaching the level of detail and quality contributed in common non-expert, volunteer-driven citizen science monitoring efforts such as Artportalen [42] (https://www.artportalen.se), OPAL [43] (http://www.opalexplorenature.org), eBird [44] (http://ebird.org) and many others [18]. In that context we explored a set of Twitter observations and ensuing conversations. Our analysis was motivated by the potential these observed ad-hoc virtual communities hold with regard to active contributions to citizen science initiatives. We discuss the analysed social media data and its “embryonic citizen science nature” with reference to the two research questions we posed in the introduction.

### 4.1 What is the type and quality of the attainable social media data, specifically in relation to comparable citizen science projects?

While identifying certain differences and gaps in the data profile, we claim that overall the analysed biodiversity observations in the form of Twitter messages and conversations do approach the type and quality of comparable citizen science data, and under consideration of the highlighted shortcomings deserve an intensified scientific and practical exploration.

A key difference between the analysed social media data and data sourced through citizen science projects is that the latter imposes a structure that is largely lacking for the analysed Twitter posts. “Rapporteurs” to the Swedish Species Observation System (Artportalen) are for example required to provide the full species name (verified against the taxonomic backbone *Dyntaxa*), geo-referenced location, the time of observation and the name of the observer [42]. However, this information is available in our analysed Twitter observations and conversations in a semi-structured format. Specifically:

- The key data item in our analysed Tweet observations are the embedded or linked images, generally accessible without restrictions, predominantly of good quality (Fig 2), thus providing sufficient detail to enable a taxonomic expert validation and determination.
- The temporal features suggest that the provided data is real-time, reported with a low latency and a good reflection of the lifecycle of the majority of the observed species, hence in line with typical biodiversity observation programmes.
- Observer and determiner information is equally available through the used Twitter accounts. The associated user profiles do not only provide background information on the contributors, but also a direct communication channel to follow up on observations or determinations.
- Precise geo-coordinates are scarce, but geo-information, albeit of lower granularity, is also available in the form of user profile locations and textual location references.
- Finally, the available information enabled 71% of determinations at the taxonomic level of “Species”, 76% of determinations were assessed as correct, although only for 16% of the determination conversations the use of scientific taxonomic names could be observed.

While acknowledging the lower quality level, we argue that there is thus only a technical rather than a conceptual challenge to utilise this data, possibly by feeding it into existing citizen science portals like Artportalen. The most notable challenges are the current lack of high-quality and reliable geo-location information as well as the level of taxonomic detail.

With regard to the first challenge, we find however that with little effort on the part of the Tweet authors the majority of observations could come with exact geo-coordinates: more than 2/3 of postings are apparently submitted from mobile devices which can be assumed to have GPS functionality, hence allow the provisioning of geo-tags; furthermore, more than half of the Tweet authors in our dataset already had their Twitter profiles geo-enabled. Thus, if users could be encouraged—for example by organizers of citizen science projects—to actively contribute observations, the utilised devices, applications and social media settings would suffice to guarantee a high degree of detailed and reliable geo-information which would not require any regular manual intervention by the user, but could possibly be of even higher quality than manually contributed data on certain citizen science platforms. While this observation is encouraging from a technical perspective, we have to take it with the caveat that we can only speculate about the reason for the surprising mismatch between the large share of geo-enabled user profiles and the lack of geo-coordinates.

The second challenge concerns the quality of taxonomic determinations. Artportalen requires observations to be reported at species level and with full scientific names. Especially the latter is not matched by our social media sample. We still argue that the quality of the recorded determinations can be judged as fairly good considering the casual conversational context and primary background of the users. Continuous technical advancements, i.e. more powerful cameras in mobile devices, will in future very likely also contribute to better quality images and determinations. Moreover, we could possibly expect contributions of higher quality, greater detail and using scientific terminology if contributors knew that they were submitting determinations to a biodiversity monitoring project. Finally, in the case of multiple (conflicting) determinations, these conversations capture a determination process in addition to determination result, which represents interesting meta-data in itself and deserves a broader and more detailed exploration with larger samples.

### 4.2 What potential do these ad-hoc social media communities hold in engaging actively with citizen science projects?

We claim that the posted biodiversity observations and ensuing determination conversations clearly match typical data collection and interpretation activities in citizen science projects [36], the data is comparable to that collected in citizen science projects and the contributor profiles hint at a large pool of contributors previously not engaged in citizen science, thus showing significant potential should the participants in our study be encouraged to graduate from a passive to an active citizen science status.

While we were not able to address those Twitter users directly and thus had to employ an indirect approach to elucidate the likely motivations, we can infer some triggers and motivations based on specific Tweet samples. In some cases the motivations were of practical nature, such as questions about the impact of a species on gardening plants and possible remedies, mostly however the basic desire for knowledge, an interest in learning what species an observation (often with a distinctive appearance) belonged to and in some cases the authors of the Tweets seemed to be motivated by a sense of discovery as indicated by for example enquiries about the potential rarity of a species. Similarly, determination providers appear to enjoy sharing their knowledge with others, and in some cases their comments and questions and the sharing of supplementary information suggested that they may also be motivated by an educational element of their participation.

Our results indicate that posted biodiversity observations and requests for determinations receive significant interest and active participation from within a Tweet author’s network (Table 2), which suggests that there is a notable implicit community detectable around these types of casual biodiversity observations. At the same time we have to note however, that the observable communities per Tweet are comparatively small; the majority of conversations receive one or two determination replies (Fig 7) and few determination conversations have more than two determinations including discussions around alternative determinations (Fig 6). While our results suggest only a small proportion of true experts in these networks, this does not necessarily imply that there is also a small share of people able or willing to reply a determination request. This could equally be attributed to conversational etiquette (i.e. it is unlikely that a user contributes a concurring opinion if the question has already been answered) rather than the number of knowledgeable potential contributors in a Twitter user’s network.

This is further supported by our categorisation of the author types: it is notably users who are not active citizen scientists, amateur biologists or domain professionals with a formal biological education that contribute observations and provide determinations (Fig 10), and non-experts or general nature enthusiasts communicating with each other (Fig 11) account for the majority of conversation replies producing determinations with a high correctness (Table 3).

In combination with the observed latency in *tweeting* the captured images, which indicates an interest in the shared observations that extends beyond the moment when the Tweet authors casually take a photo, we argue that this suggests the presence of a large pool of contributors that are currently not actively participating in formal monitoring activities, but could possibly be mobilised to regularly and actively contribute to biodiversity monitoring when such an activity involves interaction patterns comparable to the informal activities analysed here, which is the case for many citizen science biodiversity monitoring programmes.

Exact quantifications of the potential size of these embryonic citizen science communities, the mobilisation potential and the potential number of additional biodiversity observations sourced through these communities will require not only larger samples, but also an engagement with the analysed communities through direct surveys. Precise estimates are further complicated by the lack of exact numbers on the actual sample coverage of Tweets obtained through the public Twitter APIs in general and require computationally more resource-intensive directions to improve the thematic, geographic and temporal coverage and access to this data. Finally, in estimating the potential number of observations and contributors we have to take into account other social media channels as well, such as Facebook, Flickr or Instagram, and would have to include other languages and regions rather than the exclusively English language search terms used for this study.

The abundance of social media channels, users and data highlights not only the potential of this information source but also the need for suitable automation strategies in operational systems. A largely automated approach will be crucial in order to limit the required human validation effort and thus arrive at feasible costs for the extraction of relevant biodiversity information from this vast stream of social media data. Tested “Big Data” processing technologies, tools and infrastructures are however readily available [45] and can be combined with suitable filtering strategies and workflows to facilitate an economical sourcing of digital biodiversity information: data initially obtained using broad search terms (e.g. “anyone know that species”) can for example first be automatically filtered by meta-data (such as availability of geo-information or type of attached media). Depending on the data collection objective the processed data could be further filtered by pre-defined specific terms such as references to certain species, families or localities; high-volume processing approaches could directly tie into formal species databases—such as GBIF (http://www.gbif.org)—as sources of species meta-data and applicable terms for filtering (i.e. common and scientific species names). A thus sufficiently automatically filtered dataset could then be efficiently addressed in a final verification step by crowdsourcing efforts, as demonstrated by related examples in the health (e.g. http://www.crowdbreaks.com), biodiversity monitoring (e.g. http://www.ispotnature.org) and natural history domain (e.g. [46]). The automated filtering will thus significantly limit the necessary human effort, should make the cost in utilising this information feasible and result in validated data samples that could serve as training sets for further automation advances.

## 5 Conclusions

Biodiversity observations posted on Twitter and conversations with taxonomic determinations triggered by those posts appear to provide a rich, real-time data source of good quality and containing core characteristics of comparable data provided in related citizen science projects.

We can state that observational data characteristics of the *tweeted* observations and the triggered determination conversations show all elements that would be found in comparable citizen science project data. The reporting latency is low, images provide a reliable determination basis leading to conversations that produce determinations of good quality and have to offer interesting additional meta-data. The lack of detailed and reliable geo-location information stands out as a significant weakness though. We elaborated however that there is reason to believe that this could easily be alleviated. In addition, a unique feature of Twitter or similar social media tools as a data source for ecological observations is that they come with a communication channel built in, thus if the observations and determinations were to be used as monitoring data, the associated social media accounts offer a convenient way to immediately and directly follow up with the users providing the original observations.

Generally, we can conclude that a large pool of individuals with access to GPS-enabled mobile devices, no current documented but apparently more than casual interest in biodiversity observations are actively carrying these biodiversity observations into their respective social media networks, and could thus make an important active contribution to general or targeted citizen science biodiversity monitoring initiatives, both in providing and validating observations. Hence, in terms of the activity type, the contributed data and the type of participants the analysed Twitter conversations may well be termed “embryonic citizen science communities”, which merit a further exploration and have to offer practical applications for ecological monitoring and citizen science activities, if combined with suitable “Big Data” automation and processing technologies.

## Supporting Information

S1 File List of phrases used to identify Tweets that qualify as determination requests. (DOCX)

S2 File List of the 215 analysed Tweets. In accordance with Twitter terms and policies on data sharing the Tweet information is limited to the Tweet identifiers.
(CSV)

## References

[1] Xu K, Li J, Song Y. Identifying valuable customers on social networking sites for profit maximization. Expert Syst Appl. 2012;39: 13009–13018. 10.1016/j.eswa.2012.05.098

[2] Bollen J, Mao H, Zeng X. Twitter mood predicts the stock market. J Comput Sci. 2011;2: 1–8. 10.1016/j.jocs.2010.12.007

[3] Sobkowicz P, Kaschesky M, Bouchard G. Opinion mining in social media: Modeling, simulating, and forecasting political opinions in the web. Gov Inf Q. 2012;29: 470–479. 10.1016/j.giq.2012.06.005

[4] Crooks A, Pfoser D, Jenkins A, Croitoru A, Stefanidis A, Smith D, et al Crowdsourcing urban form and function. Int J Geogr Inf Sci. Taylor & Francis; 2015; 1–22. 10.1080/13658816.2014.977905

[5] Vieweg S, Hughes AL, Starbird K, Palen L. Microblogging during two natural hazards events: what twitter may contribute to situational awareness. Proceedings of the 28th international conference on Human factors in computing systems—CHI ‘10. New York, New York, USA: ACM Press; 2010. p. 1079. 10.1145/1753326.1753486

[6] Qu Y, Huang C, Zhang P, Zhang J. Microblogging after a major disaster in China: a case study of the 2010 Yushu earthquake. Proceedings of the ACM 2011 conference on Computer supported cooperative work—CSCW ‘11. New York, New York, USA: ACM Press; 2011. pp. 25–34. 10.1145/1958824.1958830

[7] Sakaki T, Okazaki M, Matsuo Y. Earthquake Shakes Twitter Users : Real-time Event Detection by Social Sensors. WWW ‘10 Proceedings of the 19th international conference on World wide web. ACM; 2010. pp. 851–860. 10.1145/1772690.1772777

[8] Crooks A, Croitoru A, Stefanidis A, Radzikowski J. #Earthquake: Twitter as a Distributed Sensor System. Trans GIS. 2013;17: 124–147. 10.1111/j.1467-9671.2012.01359.x

[9] Mykhalovskiy E, Weir L. The Global Public Health Intelligence Network and early warning outbreak detection: a Canadian contribution to global public health. Can J public Heal Rev Can santé publique. 2006;97: 42–4. Available: http://www.jstor.org/stable/41994676 10.1007/BF03405213 PMC6976220 16512327

[10] Salathé M, Bengtsson L, Bodnar TJ, Brewer DD, Brownstein JS, Buckee C, et al Digital Epidemiology. Bourne PE, editor. PLoS Comput Biol. 2012;8: e1002616 10.1371/journal.pcbi.1002616 22844241 PMC3406005

[11] Gomide J, Veloso A, Meira W, Almeida V, Benevenuto F, Ferraz F, et al. Dengue surveillance based on a computational model of spatio-temporal locality of Twitter. Proceedings of the ACM WebSci’11, June 14–17 2011, Koblenz, Germany. 2011. pp. 1–8. 10.1145/2527031.2527049

[12] De Longueville B, Smith RS, Luraschi G. “OMG, from here, I can see the flames!”: a use case mining Location Based Social Networks to acquire spatio-temporal data on forest fires. Proceedings of the 2009 International Workshop on Location Based Social Networks—LBSN ‘09. New York, New York, USA: ACM Press; 2009. pp. 73–80. 10.1145/1629890.1629907

[13] Galaz V, Crona B, Daw T, Bodin Ö, Nyström M, Olsson P. Can web crawlers revolutionize ecological monitoring? Front Ecol Environ. Ecological Society of America; 2010;8: 99–104. 10.1890/070204

[14] Daume S, Albert M, von Gadow K. Forest monitoring and social media–Complementary data sources for ecosystem surveillance? For Ecol Manage. 2014;316: 9–20. 10.1016/j.foreco.2013.09.004

[15] Cha Y, Stow CA. Mining web-based data to assess public response to environmental events. Environ Pollut. 2015;198: 97–9. 10.1016/j.envpol.2014.12.027 25577650

[16] Malcevschi S, Marchini A, Savini D, Facchinetti T. Opportunities for Web-Based Indicators in Environmental Sciences. Perc M, editor. PLoS One. 2012;7: e42128 10.1371/journal.pone.0042128 22905118 PMC3419708

[17] Stafford R, Hart AG, Collins L, Kirkhope CL, Williams RL, Rees SG, et al Eu-social science: the role of internet social networks in the collection of bee biodiversity data. PLoS One. 2010;5: e14381 10.1371/journal.pone.0014381 21179423 PMC3003702

[18] Silvertown J. A new dawn for citizen science. Trends Ecol Evol. 2009;24: 467–471. 10.1016/j.tree.2009.03.017 19586682

[19] Newman G, Wiggins A, Crall A, Graham E, Newman S, Crowston K. The future of citizen science: emerging technologies and shifting paradigms. Front Ecol Environ. Ecological Society of America; 2012;10: 298–304. 10.1890/110294

[20] Biggs R, Carpenter SR, Brock WA. Turning back from the brink: detecting an impending regime shift in time to avert it. Proc Natl Acad Sci U S A. 2009;106: 826–31. 10.1073/pnas.0811729106 19124774 PMC2630060

[21] Cooper CB, Dickinson J, Phillips T, Bonney R. Citizen science as a tool for conservation in residential ecosystems. Ecol Soc. 2007;12 Available: http://www.ecologyandsociety.org/vol12/iss2/art11/

[22] Kaplan AM, Haenlein M. Users of the world, unite! The challenges and opportunities of Social Media. Bus Horiz. 2010;53: 59–68. 10.1016/j.bushor.2009.09.003

[23] Smith A, Brenner J. Twitter Use 2012. Pew Research Center’s Internet & American Life Project. [Internet]. 2012. Available: http://pewinternet.org/Reports/2012/Twitter-Use-2012.aspx

[24] Wiggins A, Newman G, Stevenson RD, Crowston K. Mechanisms for Data Quality and Validation in Citizen Science. 2011 IEEE Seventh International Conference on e-Science Workshops. IEEE; 2011. pp. 14–19. 10.1109/eScienceW.2011.27

[25] Crall AW, Newman GJ, Stohlgren TJ, Holfelder KA, Graham J, Waller DM. Assessing citizen science data quality: an invasive species case study. Conserv Lett. 2011;4: 433–442. 10.1111/j.1755-263X.2011.00196.x

[26] See L, Comber A, Salk C, Fritz S, van der Velde M, Perger C, et al Comparing the quality of crowdsourced data contributed by expert and non-experts. Preis T, editor. PLoS One. Public Library of Science; 2013;8: e69958 10.1371/journal.pone.0069958 23936126 PMC3729953

[27] Butt N, Slade E, Thompson J, Malhi Y, Riutta T. Quantifying the sampling error in tree census measurements by volunteers and its effect on carbon stock estimates. Ecol Appl. Ecological Society of America; 2013;23: 936–943. 10.1890/11-2059.1 23865241

[28] Smith AM, Lynn S, Sullivan M, Lintott CJ, Nugent PE, Botyanszki J, et al Galaxy Zoo Supernovae. Mon Not R Astron Soc. 2011;412: 1309–1319. 10.1111/j.1365-2966.2010.17994.x

[29] Hochachka WM, Fink D, Hutchinson RA, Sheldon D, Wong W-K, Kelling S. Data-intensive science applied to broad-scale citizen science. Trends Ecol Evol. 2012;27: 130–7. 10.1016/j.tree.2011.11.006 22192976

[30] van Strien AJ, van Swaay CAM, Termaat T. Opportunistic citizen science data of animal species produce reliable estimates of distribution trends if analysed with occupancy models. Devictor V, editor. J Appl Ecol. 2013;50: 1450–1458. 10.1111/1365-2664.12158

[31] Reichman OJ, Jones MB, Schildhauer MP. Challenges and Opportunities of Open Data in Ecology. Science (80-). 2011;331 10.1126/science.1197962 21311007

[32] Sheppard SA, Wiggins A, Terveen L. Capturing quality: retaining provenance for curated volunteer monitoring data. Proceedings of the 17th ACM conference on Computer supported cooperative work & social computing—CSCW ‘14. ACM Press; 2014. pp. 1234–1245. 10.1145/2531602.2531689

[33] Otegui J, Ariño AH, Encinas MA, Pando F. Assessing the Primary Data Hosted by the Spanish Node of the Global Biodiversity Information Facility (GBIF). Raghava GPS, editor. PLoS One. 2013;8: e55144 10.1371/journal.pone.0055144 23372828 PMC3555939

[34] Crowl TA, Crist TO, Parmenter RR, Belovsky G, Lugo AE. The spread of invasive species and infectious disease as drivers of ecosystem change. Front Ecol Environ. 2008;6: 238–246. 10.1890/070151

[35] Shirk JL, Ballard HL, Wilderman CC, Phillips T, Wiggins A, Jordan R, et al Public Participation in Scientific Research: a Framework for Deliberate Design. Ecol Soc. 2012;17: art29. 10.5751/ES-04705-170229

[36] Wiggins A, Crowston K. Goals and Tasks: Two Typologies of Citizen Science Projects. 2012 45th Hawaii International Conference on System Sciences. IEEE; 2012. pp. 3426–3435. 10.1109/HICSS.2012.295

[37] Daume S. Ecoveillance (Social media analysis web platform) [Internet]. 2012. Available: http://www.ecoveillance.org

[38] Lindsey Kuper (@lindsey). “Saw this beautiful iridescent green bug today. Anyone know what it is? https://www.flickr.com/photos/lindseykuper/14360920608/ …”, 30 June 2014, 8:04 pm. Tweet. [Internet]. 2014. Available: https://twitter.com/lindsey/status/483808338015047680

[39] Croitoru A, Crooks A, Radzikowski J, Stefanidis A, Vatsavai RR, Wayant N. Geoinformatics and Social Media: A New Big Data Challenge In: Karimi HA, editor. Big Data Techniques and Technologies in Geoinformatics. Boca Raton, FL: CRC Press; 2014 pp. 207–232.

[40] Darling E, Shiffman D, Côté I, Drew J. The role of Twitter in the life cycle of a scientific publication. Ideas in Ecology and Evolution. 2013 10.4033/iee.2013.6.6.f

[41] Lindenmayer DB, Likens GE. The science and application of ecological monitoring. Biol Conserv. Elsevier Ltd; 2010;143: 1317–1328. 10.1016/j.biocon.2010.02.013

[42] Gärdenfors U, Jönsson M, Obst M, Wremp AM, Kindvall O, Nilsson J. Swedish LifeWatch ─ a biodiversity infrastructure integrating and reusing data from citizen science, monitoring and research. Hum Comput. 2014;1 10.15346/hc.v1i2.6

[43] Davies L, Bell JNB, Bone J, Head M, Hill L, Howard C, et al Open Air Laboratories (OPAL): a community-driven research programme. Environ Pollut. 2011;159: 2203–10. 10.1016/j.envpol.2011.02.053 21458125

[44] Wood C, Sullivan B, Iliff M, Fink D, Kelling S. eBird: engaging birders in science and conservation. PLoS Biol. 2011;9: e1001220 10.1371/journal.pbio.1001220 22205876 PMC3243722

[45] Hu H, Wen Y, Chua T-S, Li X. Toward Scalable Systems for Big Data Analytics: A Technology Tutorial. IEEE Access. IEEE; 2014;2: 652–687. 10.1109/ACCESS.2014.2332453

[46] Hill A, Guralnick R, Smith A, Sallans A, Rosemary Gillespie, Denslow M, et al The notes from nature tool for unlocking biodiversity records from museum records through citizen science. Zookeys. 2012; 219–33 10.3897/zookeys.209.3472 PMC3406478 22859890

