# Supplementary material for: “Anyone Know What Species This Is?” – Twitter Conversations as Embryonic Citizen Science Communities
Source: PLoS One. 2016 Mar 11;11(3):e0151387. doi: 10.1371/journal.pone.0151387 (PMC4788454; doi:10.1371/journal.pone.0151387)
Supplement: S1 File — (DOCX) [file pone.0151387.s001.docx]

| **Determination request phrases** |
| --- |
| “anyone know what”  “anybody know what”  “anyone know which”  “anybody know which”  “what this is”  “what species”  “what is this”  “what kind it is”  “know what kind of” |
